# Associations between COVID-19-related changes in the psychosocial work environment and mental health

**DOI:** 10.1177/14034948231160633

**Published:** 2023-03-24

**Authors:** Sandra Blomqvist, Marianna Virtanen, Hugo Westerlund, Linda L. Magnusson Hanson

**Affiliations:** 1 Stress Research Institute at Department of Psychology, Stockholm University, Sweden; 2 School of Educational Sciences and Psychology, University of Eastern Finland, Finland

**Keywords:** COVID-19, psychosocial work environment, work stress, employment insecurity, loneliness, anxiety, depression, psychological distress

## Abstract

**Background:**

Individuals’ lives have been substantially affected by the COVID-19 pandemic. We aimed to describe changes in psychosocial work environment and mental health and to investigate associations between job insecurity and mental ill-health in relation to changes in other psychosocial work factors, loneliness and financial worries.

**Methods:**

A sub-sample of individuals from the eighth Swedish Longitudinal Occupational Survey of Health answered a web-based survey in early 2021 about current and pandemic-related changes in health, health behaviours, work and private life. We investigated participants working before the pandemic (*N*=1231) in relation to standardised measures on depression, anxiety and loneliness, together with psychosocial work factors, in descriptive and logistic regression analyses.

**Results:**

While 9% reached the clinical threshold for depression and 6% for anxiety, more than a third felt more worried, lonelier or in a low mood since the start of the pandemic. Two per cent had been dismissed from their jobs, but 16% experienced workplace downsizings. Conditioning on socio-demographic factors and prior mental-health problems, the 8% experiencing reduced job security during the pandemic had a higher risk of anxiety, but not of depression, compared to employees with unaltered or increased job security. Loneliness and other psychosocial work factors explained more of the association than objective measures of job insecurity and financial worries.

**Conclusions:**

Reduced job security during the COVID-19 pandemic seems to have increased the risk of anxiety among individuals with a strong labour market attachment, primarily via loneliness and other psychosocial work factors. This illustrates the potentially far-reaching effects of the pandemic on mental health in the working population.

## Introduction

In March 2020, the World Health Organization declared the spread of SARS-CoV-2 a global pandemic. The pandemic and strategies to contain the spread of the virus generally changed people’s private and working lives markedly and abruptly. Globally, the unemployment rate increased by 1.1 percentage points to 6.5%, and the number of hours worked dropped considerably as workers were furloughed or received reduced working hours [1].

Sweden has frequently been highlighted in international news regarding its approach to the pandemic [2]. Compared to the other Nordic countries, Sweden stands out in terms of both high numbers of COVID-19 cases and fatality per capita [3]. Sweden primarily relied on voluntariness, individual responsibility, advice and recommendations for disease prevention and control [4–6]. Public gatherings and visits to eating and drinking facilities were restricted. Employees were advised to work from home whenever possible and to avoid unnecessary travelling and close physical contact. Institutions of higher education and upper secondary education were advised to apply distance learning, while lower-grade education and preschools remained open.

From February to June 2020, Sweden’s unemployment rate increased by two percentage points to 9.2%, and average working hours dropped by almost 11% [7]. Particularly, temporary contract workers, young workers with foreign backgrounds or those in service-sector jobs were hit the most [8].

Several systematic reviews, rapid reviews and meta-analyses have shown increasing anxiety, depression and psychological distress during the COVID-19 pandemic [9,10]. Individuals employed during the pandemic have, however, reported better mental health and less loneliness than those not in employment [11]. Employees working from home during the pandemic have reported more favourable psychosocial working conditions compared to employees working on-site [12], while pandemic-related job losses, financial worries and job insecurity have been found to increase the risk of depression, emotional exhaustion and/or anxiety [13–17]. Contrary to previous findings [18], employees exposed to workplace reorganisations reported neither higher levels of distress nor worse psychosocial working conditions compared to the employees unexposed to workplace reorganisations [12].

Psychological stress from life experiences or the anticipation thereof, such as financial strain, job loss and work situation, is the most commonly assumed mechanism linking economic crises to mental ill-health [19]. Factors such as isolation and perceived loneliness, which increased during the pandemic, may also have contributed to the increased prevalence of depression, anxiety, distress and heavy drinking [20,21].

Several published studies investigating mental-health changes during the COVID-19 pandemic have relied on small convenience samples or samples from specific settings, potentially leading to selection bias and a lack of generalisability [11,13–15]. The current study aims to investigate changes in the psychosocial work environment, mental health, loneliness and health behaviours during the COVID-19 pandemic among individuals from the general working population in Sweden. Specifically, we aimed to investigate whether perceptions of changes in job insecurity during the pandemic were associated with an increased risk of depression and/or anxiety, and whether such an association could be explained by changes in other areas of life and work during the pandemic, such as psychosocial working conditions, loneliness or the economy.

## Methods

### Study participants and sampling

**Figure 1.**
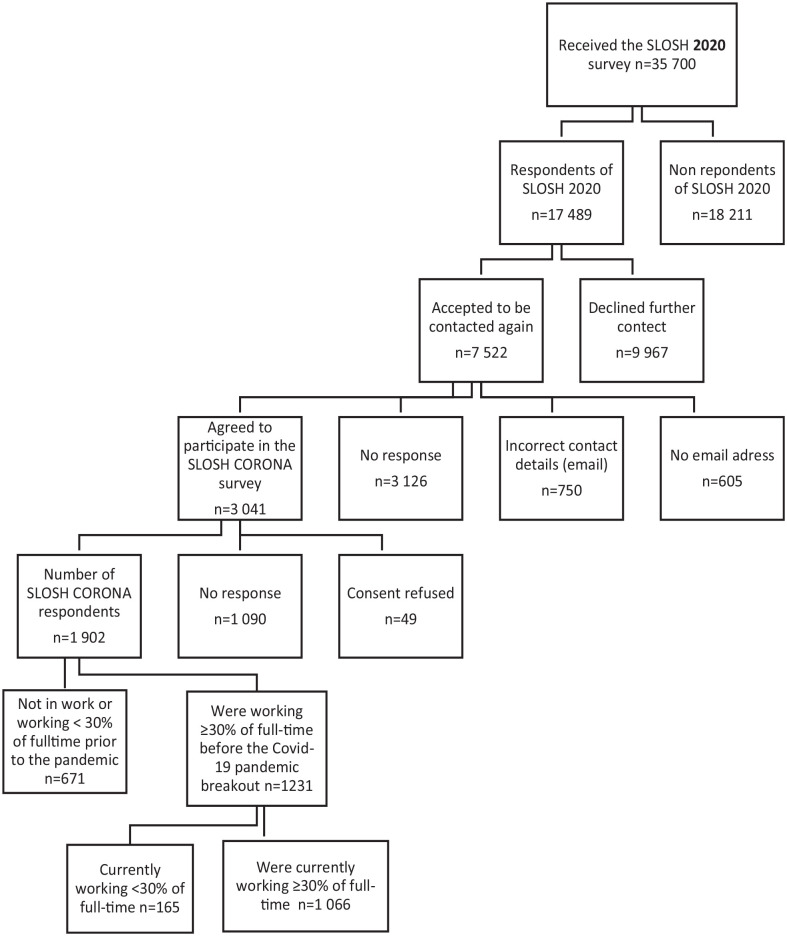
Flow chart of study participants in the SLOSH Corona survey.

This study is based on information from participants in the Swedish Longitudinal Occupational Survey of Health (SLOSH) study, targeting an initially nationally representative sample of working Swedish residents in paid work or who have left the labour market. Auxiliary to the regular bi-annual SLOSH data collection [22], the SLOSH Corona survey gathered information about work, health and health behaviours during the COVID-19 pandemic. The sample is based on respondents of the SLOSH 2020 data collection (*n*=17,489), who agreed, and provided correct contact details, to be contacted in later data collections (*n*=3041). In total, 1902 responded to the SLOSH Corona web survey in January–February 2021. In this study, we included only participants who responded that they worked at least 30% full-time (approximately 40 hours a week) before the pandemic (*N*=1231; see Figure 1). Participants were further asked whether they were currently working at least 30% full-time. If so (*n*=1066), they received questions about their current work situation; if not (*n*=165), they were only asked about health, lifestyle and transitions into economic inactivity. SLOSH Corona was given ethical permission by the Swedish Ethical Review Authority. Compared to the general SLOSH population [22], the SLOSH Corona sample was slightly older, with a higher income and education, but scored similarly on depression and anxiety, job insecurity and other socio-demographic factors.

### Covariets

Information about sex, age, income, educational level and country of birth were derived from the Longitudinal Integration Database for Health Insurance and Labour Market Studies (LISA) and civil status was obtained from the SLOSH 2020 survey.

### Employment situation

Changes in job security, total workload, psychological pressure at work, support from bosses or colleagues, degree of influence, atmosphere at work, degree of unity and collaborations were assessed by asking participants to rate any changes with regard to the following statement: ‘If you compare your employment since the start of the Corona pandemic with your employment situation as it was before the Corona pandemic, has. . . [the set of factors presented above] changed?’ Reponses were from a five-point Likert scale, ranging from 1=reduced largely to 5=increased largely. Responses were collapsed into 1=reduced, 2=unaltered, 3= increased. Job insecurity was operationalised using a dichotomous measure where 1=job security reduction and 0=no job security reduction (i.e. unaltered or increased), henceforth referred to as the insecure and the secure groups, respectively (see Supplemental Material, ‘Item information’, for further details). Participants were also asked whether or not (yes or no) they had received a notice of dismissal, furlough, worktime reduction, become unemployed, experienced reorganisations, downsizings or closure, temporarily changed work tasks or a worsened personal economy since the COVID-19 outbreak, as well as whether they had experienced bullying, violence or threats thereof or gender harassments in the past six months.

### Mental health

The nine-item Patient Health Questionnaire (PHQ-9) was used to identify individuals likely to be clinically diagnosed with depression (range=0–27; cut-off >9 for moderate to severe depression) [23]. The scale is based on diagnostic criteria from DSM-IV, showing good reliability and discriminating properties [23]. Correspondingly, the seven-item General Anxiety Disorder (GAD-7) scale, which has shown good internal consistency, procedural and construct validity [24], was used to identify respondents with symptoms of anxiety of presumed clinical relevance (range=0–21, cut-off ⩾10). We also dichotomised information on whether respondents felt more worried or in a low mood since the pandemic outbreak compared to before the pandemic (see Supplemental Material (Appendix) for details). Lastly, prior mental-health status was assessed by calculating scores of depressive symptoms according of the Symptom Checklist-core depression (SCL-CD6) scale, available in the SLOSH 2020 survey.

### Loneliness and health behaviours

Loneliness was assessed using the UCLA Loneliness Scale (short three-item T-ILS version) before and during the pandemic [25]. For the descriptive analyses, a change score was used to categorise respondents experiencing greater, less or unaltered loneliness. In the multivariate analysis, the person’s actual loneliness score during the pandemic was used. Heavy drinkers were distinguished from non-heavy drinkers using the Alcohol Use Disorders Identification Test-Consumption (AUDIT-C) [26], covering the time since the outbreak of the COVID-19 pandemic. As recommended by the Swedish Public Health Agency, we used sex-specific cut-offs considering both number of drinking occasions per week and the usual amount of alcohol intake on such an occasion. Detailed item information is available in the Supplemental Material [27].

### Statistical analysis

We conducted descriptive analyses on demographic, health- and work-related factors and health behaviours. We compared the sample to the general population in SLOSH using chi-square and independent group *t*-tests to assess its representativeness. Differences in health, work and demographics between (job) insecure and secure employees were examined using chi-square and analysis of variance tests. The association between job insecurity, anxiety and depression was investigated using logistic regression models. After the crude model (model 1), we adjusted for socio-demographic factors and prior mental health (model 2), actual experiences of job loss or instability, including furlough, notice of layoff, dismissal or enforced work-time reduction (model 3), changes in other psychosocial work factors (model 4), loneliness (model 5) or personal economy (model 6, available in the Supplemental Material). Interactions between job insecurity and loneliness and support from supervisors, respectively, were assessed on the multiplicative scale by including an interaction term in the logistic model, and the additive scale by calculating the relative excess risk due to interaction (RERI) and synergy index (SI). All analyses were performed with SAS v9.4 (SAS Institute, Cary, NC).

## Results

### Characteristics of SLOSH Corona population

In total, 1231 individuals reported working ⩾30% full-time before the COVID-19 outbreak, of whom 87% (*n*=1066) also did so in January–February 2021. Among those working <30% full-time or not at all (*n*=165), 65 (5.2%) stated that they had completely or partially retired during the pandemic, and 45 (3.8%) stated that they became unemployed at some point between April 2020 and January/February 2021. Overall, more women (*n*=700; 57%) than men responded to the survey, and respondents were on average 55 years old. Most respondents (78%; *n*=947) were either married or cohabiting, and 65% had at least two years of education at the university level. Almost 9% (*n*=106) of the respondents reached the clinical threshold of depressive symptoms and 6% (*n*=69) for symptoms of anxiety. A third of the sample reported feeling low or more worried since the pandemic started; 55% reported a greater sense of loneliness, while only 3% (*n*=28) reported heavy drinking behaviours (see Table I).

**Table I.** Descriptive characteristics of participants and further by status on perceived changes in job security during the COVID-19 pandemic.

		Total (*N*=1231)		Reduced perceived job security (*N*=80)		Unchanged or improved (*N*=894)		Test of difference^a^
		*n* (*M*)	% (*SD*)	*n* (*M*)	% (*SD*)	*n* (*M*)	% (*SD*)	(*p*-Value)
Currently working ⩽30% full-time		1066	86.6					
Unemployed		45	3.7					
Retired (completely or partially)		65	5.2					
Age		55.4	11.2	50.7	10.8	55.2	11.0	**0.0005**
Women		700	56.9	42	52.5	503	56.3	0.5159
Married/cohabiting		947	77.7	61	77.2	682	76.7	0.9197
Yearly income from work		486,883	251,891	505,320	251,640	494,502	240,539	0.7011
Compulsory		37	3.0	1	1.3	27	3.0	
Upper secondary		388	31.6	24	30.0	282	31.6	
University		804	65.4	55	68.7	583	65.4	0.6083
Employed in industry affected by the COVID-19 pandemic^b^		70	5.7	7	8.8	37	4.1	0.0571
Furloughed		151	12.6	23	28.8	90	10.1	**<0.0001**
Workplace downsizing		161	15.6	43	55.1	113	12.7	**<0.0001**
Worsened personal economy		177	14.8	29	36.3	65	7.3	**<0.0001**
Total workload	Decreased	200	19.7	38	48.1	148	16.6	
	Unchanged	479	47.1	16	20.3	442	49.6	
	Increased	338	33.2	25	31.7	301	33.8	**<0.0001**
Psychological pressure	Decreased	103	10.3	11	14.5	84	9.6	
	Unchanged	526	52.6	30	39.5	478	54.3	
	Increased	371	37.1	35	46.1	318	36.1	**0.0393**
Degree of influence	Decreased	112	11.1	21	26.9	85	9.6	
	Unchanged	782	77.4	45	57.7	702	79.1	
	Increased	116	11.5	12	15.4	101	11.4	**<0.0001**
Support from supervisors	Decreased	148	14.9	28	36.8	110	12.5	
	Unchanged	658	66.4	34	44.7	611	69.6	
	Increased	185	18.7	14	18.4	157	17.9	**<0.0001**
Support from colleagues	Decreased	173	17.3	24	32.0	137	15.5	
	Unchanged	647	64.6	34	45.3	596	67.3	
	Increased	182	18.1	17	22.7	153	17.3	**0.0002**
Atmosphere at work	Worsened	388	39.5	46	66.7	321	37.1	
	Unchanged	505	51.4	19	27.5	465	53.7	
	Improved	89	9.1	4	5.8	80	9.2	**<0.0001**
Degree of unity	Worsened	358	36.3	36	51.4	302	34.7	
	Unchanged	455	46.1	23	32.9	418	48.1	
	Improved	174	17.6	11	15.7	150	17.2	**0.0157**
Degree of collaboration	Worsened	320	32.7	33	47.1	265	30.7	
	Unchanged	494	50.5	28	40.0	452	52.4	
	Improved	165	16.8	9	12.9	146	16.9	**0.0179**
Depressive symptoms (PHQ-9 cut-off)	Yes	106	8.7	8	10.0	66	7.4	0.4025
Anxiety (GAD-7 cut-off)	Yes	69	5.6	12	15.2	36	4.0	**<0.0001**
Increase in feelings of worry since pandemic outbreak		329	30.3	39	50.0	213	26.3	**<0.0001**
Increase of low mood since pandemic outbreak		396	36.2	46	58.2	267	32.7	**<0.0001**
AUDIT heavy drinking		28	2.6	2	2.9	21	2.6	0.9117
Loneliness score		6.0	2.7	7.3	2.7	5.8	2.7	**<0.0001**
Increased loneliness compared to before COVID-19 pandemic		663	55.43	58	73.4	478	53.8	**0.0008**

a Test of difference performed between groups, where 1=reduced and 0=unaltered/improved job security.

b Occupations within the 10 industries affected the most by cutbacks during the pandemic (e.g. hotel, restaurant, tourism, culture, entertainment, etc.) according to Statistics Sweden.

PHQ-9: Patient Health Questionnaire; GAD-7: General Anxiety Disorder scale.

About 13% (*n*=151) of the respondents were furloughed. Among those still working, 16% (*n*=161) were employed at an organisation that had downsized their personnel, while <2% had been dismissed or received notice. Both improvements and deteriorations in working conditions were reported (see Table I). About 8% (*n*=80) of those with information on job security (*n*=974) reported reduced job security, 20% (*n*=200) reported that their total workload had increased, 17% experienced decreasing support from colleagues and nearly 40% a worsening in the general work atmosphere. Nearly 2% experienced bullying in the past six months, and 1% had experienced violence/threats of violence and gender harassments (data not shown).

### Perceived job security since the outbreak of the COVID-19 pandemic

While we found no gender differences, those reporting that the pandemic reduced their job security were younger (51 years old) compared to those with unchanged/improved job security (55 years old; Table I).

No differences in depressive symptoms, according to PHQ-9 clinical threshold, were observed, but a larger proportion of individuals in the insecure versus the secure group reached the clinical threshold for anxiety (GAD-7; 15% (*n*=12) and 4% (*n*=36), respectively). The insecure employees more often reported increased worry or low mood (50%, 58%) compared to secure employees (26%, 33%) and scored higher on the loneliness scale (7.3 compared to 5.8). The prevalence of heavy drinking behaviours was similar, but the insecure more often reported a worsened economy than the secure group. Lastly, reduced support from colleagues (32%), supervisors (37%) and workload (48%) were also more common among the insecure than the secure employees (13%, 16% and 17%; Table I).

### Logistic regression on perceived change of job insecurity and mental-health outcomes

In the unadjusted models, reduced job security was associated with higher odds of reaching the clinical threshold of anxiety symptoms (GAD-7 scale; odds ratio (OR)=4.56, 95% confidence interval (CI) 2.26–9.21; Table II) but not the clinical threshold for depressive symptoms (PHQ-9 scale; OR=1.46, 95% CI 0.68–3.18; Table III).

**Table II.** Logistic regression models of anxiety (GAD-7) by exposure to perceived reduced JS, according to different sets of adjustments (*N*=974).

	Model 1: Crude	Model 2: Socio-demographic and baseline mental health	Model 3: Model 2+employment changes	Model 4: Model 2+psychosocial working conditions	Model 5: Model 2+perceived loneliness
	OR (95% CI)	OR (95% CI)	OR (95% CI)	OR (95% CI)	OR (95% CI)
Stable/improved JS	1.00	1.00	1.00	1.00	1.00
Reduced JS	4.56 (2.26–9.21)	3.00 (1.42–6.37)	3.81 (1.66–8.71)	2.59 (1.01–6.64)	2.20 (0.98–4.91)
Women		0.61 (0.32–1.15)	0.57 (0.30–1.09)	0.48 (0.24–0.99)	0.37 (0.18–0.76)
Age		0.97 (0.94–1.00)	0.97 (0.94–0.99)	0.97 (0.94–1.00)	0.97 (0.94–1.00)
Quartile 1		1.30 (0.51–3.30)	1.21 (0.47–3.15)	1.55 (0.55–4.35)	1.42 (0.53–3.83)
Quartile 2		1.01 (0.38–2.66)	0.88 (0.32–2.44)	1.34 (0.46–3.84)	1.11 (0.40–3.07)
Quartile 3		1.43 (0.59–3.46)	1.45 (0.59–3.53)	1.66 (0.64–4.30)	1.39 (0.55–3.49)
Mental health at baseline		2.16 (1.66–2.82)	2.17 (1.66–2.85)	2.08 (1.56–2.76)	1.95 (1.47–2.58)
Furloughed			0.68 (0.17–2.77)		
Dismissal or notice			0.73 (0.13–4.00)		
Worktime reduction			0.55 (0.11–2.75)		
Increased workload				1.17 (0.52–2.59)	
Reduced workload				0.70 (0.21–2.27)	
Increased psychological pressure				2.87 (1.27–6.49)	
Reduced psychological pressure				0.43 (0.05–3.67)	
Increased influence				0.56 (0.17–1.79)	
Reduced influence				1.11 (0.41–3.04)	
Increased support from supervisors				1.23 (0.45–3.35)	
Reduced support from supervisors				1.71 (0.69–4.26)	
Increased support from colleagues				0.75 (0.27–2.11)	
Reduced support from colleagues				1.05 (0.42–2.65)	
UCLA loneliness score					1.46 (1.28–1.66)

Statistically significant (*p*<0.05) values indicated in bold.

JS: job security; OR: odds ratio; CI: confidence interval.

**Table III.** Logistic regression models of depression (PHQ-9) by exposure to perceived reduced JS, according to different sets of adjustments (*N*=974).

	Model 1: Crude	Model 2: Socio-demographic and baseline mental health	Model 3: Model 2+employment changes	Model 4: Model 2+psychosocial working conditions	Model 5: Model 2+perceived loneliness
	OR (95% CI)	OR (95% CI)	OR (95% CI)	OR (95% CI)	OR (95% CI)
Stable/improved JS	1.00	1.00	1.00	1.00	1.00
Reduced JS	1.46 (0.68–3.18)	0.98 (0.44–2.21)	1.20 (0.51–2.80)	0.65 (0.24–1.75)	0.67 (0.29–1.56)
Women		0.82 (0.49–1.36)	0.81 (0.48–1.35)	0.65 (0.37–1.15)	0.55 (0.32–0.96)
Age		0.98 (0.96–1.01)	0.98 (0.96–1.01)	0.99 (0.96–1.01)	0.99 (0.96–1.01)
Quartile 1		0.82 (0.39–1.71)	0.78 (0.37–1.63)	0.84 (0.37–1.89)	0.88 (0.40–1.92)
Quartile 2		0.93 (0.45–1.90)	0.84 (0.40–1.75)	1.18 (0.55–2.55)	1.04 (0.49–2.19)
Quartile 3		1.11 (0.56–2.17)	1.11 (0.56–2.17)	1.17 (0.57–2.42)	1.13 (0.56–2.30)
Mental health at baseline		**1.85 (1.51–2.28)**	**1.86 (1.51–2.29)**	**1.79 (1.43–2.25)**	**1.65 (1.32–2.05)**
Furloughed			1.32 (0.49–3.55)		
Dismissal or notice			0.35 (0.04–2.96)		
Worktime reduction			0.63 (0.19–2.02)		
Increased work load				1.08 (0.57–2.03)	
Reduced work load				0.99 (0.40–2.45)	
Increased psychological pressure				**3.34 (1.74–6.43)**	
Reduced psychological pressure				0.48 (0.10–2.29)	
Increased influence				0.71 (0.30–1.69)	
Reduced influence				1.07 (0.47–2.37)	
Increased support from supervisors				1.58 (0.73–3.40)	
Reduced support from supervisors				**2.19 (1.04–4.59)**	
Increased support from colleagues				0.9 (0.41–1.99)	
Reduced support from colleagues				1.31 (0.64–2.71)	
UCLA loneliness score					**1.40 (1.27–1.55)**

Statistically significant (*p*<0.05) values indicated in bold.

The main association with symptoms of anxiety persisted, but it was attenuated when adjusting for socio-demographic factors and prior mental health (OR=3.00, 95% CI 1.42–6.37). Adjusting for job loss/instability did not reduce the risk estimate (OR=3.81, 95% CI 1.66–8.71), while adjustments for a simultaneous deterioration of other psychosocial work factors did (OR=2.59, 95% CI 1.01–6.64), in particular increasing psychological pressure and decreasing support from supervisors. Similarly, adjustment for perceived loneliness further attenuated the association (OR=2.20, 95% CI 0.98–4.91). However, the two-way interactions between job security and being alone during the pandemic and with decreasing support from supervisors, respectively, were not statistically significant, neither were the estimates of interactions on the additive scale (RERI and SI; data not shown). A worsened personal economy during the pandemic also attenuated the main estimate to some extent (see Supplemental Table S1), but this was not a statistically significant predictor.

## Discussion

We found that those who experienced a reduction in job security during the pandemic were more likely to report increasing anxiety but not depression. Consistent with other reports [21], it was also common to feel lonelier during the first year of the pandemic.

Previous studies have found COVID-19 pandemic-related job insecurity to increase the risk of both anxiety and depression [15–17]. These are often co-morbid conditions, and many suffer from both, but there are also important distinctions between anxiety and depression. While depression often is associated with adverse events from one’s past (e.g. losses), anxiety tends to be linked to concerns about potential future events [28]. Individuals with anxiety may also perceive future events to be temporally closer than those with depression [29], potentially making these events feel more impeding. In our sample, depression was more common than anxiety and more common among those who were unemployed during the pandemic compared to those who experienced job insecurity. However, full (unemployment, dismissal) or partial (noticed, furloughed) job loss was not associated with depression or anxiety. This may be explained by weak statistical power which required a crude categorisation of job loss/instability. Any associations between different types of job loss, such as unemployment and mental health, may thus have remained undetected. These findings are on the other hand consistent with another study which found no difference in psychological distress or perceived psychosocial work situation between employees who were exposed and unexposed to reorganisations [12]. Furthermore, adjustment for actual experiences of permanent or temporary job loss did not affect the association between job insecurity and mental health. This could suggest that we primarily captured mental-health consequences of affective job insecurity (i.e. a perceived fear/worry about a job loss) rather than of cognitive job insecurity (i.e. the perceived likelihood of job loss) which may be associated with job instability such as being furloughed, receiving a notice or enforced work-time reduction. This is consistent with prior research showing a stronger relationship between affective job insecurity and mental health and that worrying about a future job loss is a likely mediator for negative mental-health outcomes, in the presence of cognitive job insecurity [30].

Job insecurity was also associated with experiencing loneliness and a deterioration of other psychosocial work factors during the pandemic, which could represent possible pathways to the observed mental ill health in this group of employees. Many job insecurity and mental-health studies during the COVID-19 pandemic have been from the USA, and show that job insecurity is associated with anxiety due to worries about one’s personal economy [17]. We examined perceptions of worsened personal economy, but we found that support from colleagues and bosses, and loneliness, had a larger influence on the association between job security and anxiety. Taken together, both monetary and social pathways between job insecurity and ill-health may thereby be of importance, in accordance with Jahoda’s latent deprivation theory [31]. However, the significance of one pathway over others may vary, depending on context.

In accordance with recommendations for future research on psychosocial work and mental health [32], we examined associations between job insecurity and clinically relevant symptoms of anxiety and depression, relying on validated scales that conform to diagnostic criteria for these mental disorders. The proportion of individuals who met the clinical threshold for depression and anxiety was fairly small and comparable to the pre-pandemic prevalence of depression among people in employment [33] but lower than the prevalence in the general Swedish population before [34] and during the pandemic [14]. This could be attributed to the larger proportion of women, young individuals, foreign-born and economically inactive people included in those studies. In this study, worry and low mood was more commonly reported during compared to prior to the pandemic, but these states also tend to be common during non-pandemic times [35]. We did account for pre-existing mental-health symptoms in order to reduce the risk of reverse causality. However, we cannot eliminate that an individual’s pre-existing anxiety or depression affected his/her reporting of job insecurity. In addition, we cannot rule out common method bias, since information on the exposure, outcomes and covariates came mainly from the same data source at a cross-section of time. Furthermore, unmeasured confounding could have influenced our findings. For instance, we did not have measures for personality traits, which could at least in part explain the association between job insecurity and mental health [36], and nor did we consider the fear of being infected, which also may have contributed to anxiety and feeling depressed, alongside changes in the employment situation.

Only about 6% of our sample worked within industries most severely affected by the pandemic, and few respondents were dismissed or noticed. It was more common that participants experienced reorganisations, downsizings and furloughs. Furthermore, young workers, foreign-born and individuals on fixed-termed contracts were underrepresented in the survey, which affects the generalisability of our findings to populations with high levels of precarious employment. It is possible that those in our sample, as they had a fairly strong labour market attachments and good health, were less severely affected by personnel reductions during the pandemic. Still, the inclusion of actual downsizings, furloughs and notices, often associated with job insecurity, together with other psychosocial work factors, loneliness and drinking behaviours strengthen our study, although the single-item measure of job insecurity may have increased the risk of measurement error. This study aimed to investigate how work situations and other factors have changed during the pandemic. Thus, we asked participants how they perceived changes in job security, mood and worry. A before-and-after measurement may have been preferable, although using respondents as their own reference points could have minimised the risk of bias by what is perceived as reasonable given one’s circumstances (e.g. age) [37]. Ceiling and categorisation problems may also have been reduced. However, as the questions regarding changes in mood or worrying have not been validated and may overlap with criterion variables, we restricted the multivariate analysis to outcomes based on the established scales on depression (PHQ-9) and anxiety (GAD-7).

To conclude, relatively few individuals in this study reported symptoms of anxiety and depression of clinical relevance, while an increase in loneliness, low mood and worry during the pandemic was common. Still, this study lends support that there is an association between a reduction in job insecurity and anxiety. The findings are relevant for clinicians meeting and treating individuals with symptoms of anxiety and for policymakers formulating job-retention schemes during economic contraction and policies for preventing work-related illness during pandemics.

## Supplemental Material

sj-docx-1-sjp-10.1177_14034948231160633 – Supplemental material for Associations between COVID-19-related changes in the psychosocial work environment and mental health Click here for additional data file. Supplemental material, sj-docx-1-sjp-10.1177_14034948231160633 for Associations between COVID-19-related changes in the psychosocial work environment and mental health by Sandra Blomqvist, Marianna Virtanen, Hugo Westerlund and Linda L. Magnusson Hanson in Scandinavian Journal of Public Health

sj-docx-2-sjp-10.1177_14034948231160633 – Supplemental material for Associations between COVID-19-related changes in the psychosocial work environment and mental health Click here for additional data file. Supplemental material, sj-docx-2-sjp-10.1177_14034948231160633 for Associations between COVID-19-related changes in the psychosocial work environment and mental health by Sandra Blomqvist, Marianna Virtanen, Hugo Westerlund and Linda L. Magnusson Hanson in Scandinavian Journal of Public Health
