# Supplementary material for: Associations between COVID-19-related changes in the psychosocial work environment and mental health
Source: Scand J Public Health. 2023 Mar 24;51(5):664–72. doi: 10.1177/14034948231160633 (PMC10040465; doi:10.1177/14034948231160633)
Supplement: sj-docx-1-sjp-10.1177_14034948231160633 – Supplemental material for Associations between COVID-19-related changes in the psychosocial work environment and mental health [file sj-docx-1-sjp-10.1177_14034948231160633.docx]

| **Supplementary Table 1. Logistic regression model onreduced job insecurity in relation to anxiety and depression, adjusting for a worsened personal economy (n=974)** | | |
| --- | --- | --- |
|  | **Gad-7** | **Phq-9** |
|  | OR (95% CI) | OR (95% CI) |
|  |  |  |
| Stable/improved JS | 1.00 | 1.00 |
| Reduced Job security | 2,47 (1,06-5,76) | 0,84 (0,35-2,02) |
| Women | 0,62 (0,33-1,18) | 0,81 (0,48-1,35) |
| Age | 0,97 (0,94-1,00) | 0,98 (0,96-1,01) |
| Quartile 1 | 1,24 (0,49-3,19) | 0,84 (0,40-1,79) |
| Quartile 2 | 0,95 (0,35-2,53) | 0,95 (0,46-1,98) |
| Quartile 3 | 1,40 (0,58-3,38) | 1,15 (0,58-2,29) |
| Mental health at baseline | 2,17 (1,66-2,83) | 1,88 (1,52-2,31) |
| Worsened economy | 1,62 (0,66-4,01) | 1,52 (0,69-3,35) |
