# Supplementary material for: Associations between COVID-19-related changes in the psychosocial work environment and mental health
Source: Scand J Public Health. 2023 Mar 24;51(5):664–72. doi: 10.1177/14034948231160633 (PMC10040465; doi:10.1177/14034948231160633)
Supplement: sj-docx-2-sjp-10.1177_14034948231160633 – Supplemental material for Associations between COVID-19-related changes in the psychosocial work environment and mental health [file sj-docx-2-sjp-10.1177_14034948231160633.docx]

Appendix 1,

Description of Study Variables

Survey questions and grouping of response alternatives

**Job insecurity**

*If you compare your employment since the start of the Corona pandemic with your employment situation as it was before the Corona pandemic with regard to …- Job security?*

| 1 = Decreased a lot  2 = Decreased slightly  3 = Unchanged  4 = Increased slightly  5 = Increased a lot  6 = Don’t know/not applicable | 1= Exposed to greater job insecurity since start of pandemic, (the insecure group) |
| --- | --- |
|  |  |
|  | 0= *Not* exposed to greater job insecurity since start of pandemic, (the secure group) |
|  | Missing |

**Other psychosocial work factors**

*If you compare your employment since the start of the Corona pandemic with your employment situation as it was before the Corona pandemic with regard to… -Total workload? –Psychological pressure at work? -* *Degree of influence? -Support from supervisors? -Support from colleagues?*

| 1 = Decreased a lot  2 = Decreased slightly  3 = Unchanged  4 = Increased slightly  5 = Increased a lot  6 = Don’t know/not applicable | 1=A *Decrease* in the respective psychosocial work factor |
| --- | --- |
|  | 2= Psychosocial work factor remained *Unchanged* |
|  | 3=An *Increase* in the respective psychosocial work factor |
|  | Missing |

*If you compare your employment since the start of the Corona pandemic with your employment situation as it was before, how are things changed with regard to… -Workplace atmosphere? –Degree of unity at work? – Collaborating at work?*

| 1 = A lot worse  2 = Slightly worse  3 = Unchanged  4 = Improved slightly  5 = Improved a lot  6 = Don’t know/not applicable | 1=A *worsening* in the respective psychosocial work factor |
| --- | --- |
|  | 2= Psychosocial work factor remained *Unchanged* |
|  | 3=An *improvement* in the respective psychosocial work factor |
|  | Missing |

*Have you in the past 6 months been exposed to…- Workplace bullying? – Workplace gender harassments? - Violence or threat of violence at work?*

| 1 = Never  2 = Very occasionally  3 = One or more times/month  4 = One or more times/week | 0= No |
| --- | --- |
|  | 1=Yes |

**Symptoms of distress or low wellbeing**

*If you compare your health/wellbeing since the start of the Corona pandemic with how it was before the Corona pandemic with regard to… -Low mood? –Nervousness and inner worry?*

| 1 = Decreased a lot  2 = Decreased slightly  3 = Unchanged  4 = Increased slightly  5 = Increased a lot  6 = Don’t know/not applicable | 0=No change or improved health/wellbeing |
| --- | --- |
|  | 1= Worse health/wellbeing |
|  | Missing |

**Heavy drinking**

Men scoring 6 or higher are considered having a hazardous drinking behavior. The corresponding cut-off for women is a score of 5 or higher.

*How often have you been drinking alcohol since the Covid-19 pandemic started?*

| 0 = Never  1 = Once a month or more seldom  2 = 2-4 times a month  3 = 2-3 times a week  4 = 4 times a week or more |
| --- |
|  |
|  |

*How many “glasses” (see below) do you usually have a typical day when you have been drinking alcohol during the Covid-19 pandemic? By one “glass” we mean 50 cl of beer alc. 3,5%, 33 cl of beer alc. 5%, a glass of red or white wine, a small glass of fortified wine or 4 cl of liquor e.g., whiskey*

| 0 = 1-2 glasses  1 = 3-4 glasses  2 = 5-6 glasses  3 = 7-9 glasses  4 = 10 glasses or more |
| --- |
|  |
|  |
